# A Radial Glia Gene Marker, Fatty Acid Binding Protein 7 (FABP7), Is Involved in Proliferation and Invasion of Glioblastoma Cells

**DOI:** 10.1371/journal.pone.0052113

**Published:** 2012-12-21

**Authors:** Antonella De Rosa, Serena Pellegatta, Marco Rossi, Patrizia Tunici, Letizia Magnoni, Maria Carmela Speranza, Federico Malusa, Vincenzo Miragliotta, Elisa Mori, Gaetano Finocchiaro, Annette Bakker

**Affiliations:** 1 Siena Biotech Spa, Siena, Italy; 2 Unit of Molecular Neuro-Oncology, Neurological Institute C. Besta, Milan, Italy; 3 Department of Experimental Oncology, European Institute of Oncology, Milan, Italy; 4 Department of Animal Pathology, Prophylaxis and Food Hygiene, University of Pisa, Pisa, Italy; NIH/NCI, United States of America

## Abstract

Glioblastoma multiforme (GBM) is among the most deadly cancers. A number of studies suggest that a fraction of tumor cells with stem cell features (Glioma Stem-like Cells, GSC) might be responsible for GBM recurrence and aggressiveness. GSC similarly to normal neural stem cells, can form neurospheres (NS) in vitro, and seem to mirror the genetic features of the original tumor better than glioma cells growing adherently in the presence of serum. Using cDNA microarray analysis we identified a number of relevant genes for glioma biology that are differentially expressed in adherent cells and neurospheres derived from the same tumor. Fatty acid-binding protein 7 (FABP7) was identified as one of the most highly expressed genes in NS compared to their adherent counterpart. We found that down-regulation of FABP7 expression in NS by small interfering RNAs significantly reduced cell proliferation and migration. We also evaluated the potential involvement of FABP7 in response to radiotherapy, as this treatment may cause increased tumor infiltration. Migration of irradiated NS was associated to increased expression of FABP7. In agreement with this, in vivo reduced tumorigenicity of GBM cells with down-regulated expression of FABP7 was associated to decreased expression of the migration marker doublecortin. Notably, we observed that PPAR antagonists affect *FABP7* expression and decrease the migration capability of NS after irradiation. As a whole, the data emphasize the role of FABP7 expression in GBM migration and provide translational hints on the timing of treatment with anti-FABP7 agents like PPAR antagonists during GBM evolution.

## Introduction

Gliomas are the most common primary malignancy in the central nervous system (CNS). These tumors exhibit histological resemblance to glial cells. They are classified into WHO grades I to IV [1] with grade III and grade IV (glioblastoma multiforme, GBM) representing the more malignant tumors.

Despite improvements in therapeutic strategies the median survival times of high grade gliomas remain low [2]. The development of novel, more efficacious therapies for this highly complex disease are therefore required.

Recent findings have paved the way towards a better understanding of the biology of glioblastoma. In particular, it has been suggested that many tumors contain a subpopulation of cancer cells possessing stem cell properties. These “cancer stem-like cells” were reported to contribute to invasion and chemoresistance of glioblastoma tumors [3], [4]. They are defined as cells that demonstrate stem cell properties (self renewal/multi differentiation capacity), grow as neurospheres, and are functionally associated with increased aggressiveness in terms of invasion/reduced differentiation (more flexible to adapt to different environments), and increased chemoresistance. More importantly, when injected in vivo they are able to partially recapitulate the phenotype of the tumor of the patient from which they are derived [5]. Although there is no unanimity around the exact role and nature of cancer stem cells, many studies converge in showing that under specific culture conditions GBM cells tend to form spheres that contain stem-like cells [6]–[8]. Whether these cells are pure cancer stem cells remains a matter of debate and in the absence of markers that differentiate stem from non-stem cells [9], [10] the question will remain unanswered. However, Lee et al. [11] have demonstrated that cells derived from patient tumors cultured in stem-promoting conditions as neurospheres, maintain the pheno- and geno-type of the original tumor better than the same cells cultured as adherent cells under classical, serum-containing conditions. Also in the current study we find that neurospheres, display typical characteristics (invasion, migration, proliferation) of the clinically relevant GBM much better than their adherent counterpart.

In order to identify targets that may have more clinical relevance than those identified in adherent cells, we performed microarray experiments on adherent and sphere-growing cells from patient-derived tumors. The radial glia gene *FABP7* came out as one of the most differentially expressed genes between neurospheres and adherent cells.

Fatty acid binding protein 7 (FABP7) also known as brain lipid binding protein, (BLBP), is a human gene mapping to chromosome 6 q22–23. It is a member of the FABP family, consisting of structurally related proteins that have specific cell, tissue, and development patterns of expression. *FABP7* was first isolated from a foetal brain cDNA library, and the transcript was detected in adult human brain and skeletal muscle but not in other normal adult tissue [12]. Also in brain tissue human *FABP7* gene is expressed more abundantly at an immature stage of the brain than after maturation [12].

Potential FABP7 binding partners recently described by Oeemig et al. [13] are docosahexaenoic (DHA), oleic, linoleic, and elaidic acid.

Generally, FABP proteins are involved in the uptake and intracellular trafficking of fatty acids, bile acids, and retinoids, as well as in cell signalling, gene transcription, cell growth, and differentiation. In radial glial cells, FABP7 plays a role in the establishment of the radial glial system required for neuronal migration [14]. In addition, Taylor et al. [15] found that all ependymoma derived tumor spheres displayed a CD133+/Nestin+/RC2+/BLBP+ immunophenotype similar to that of radial glia cells.

In glioblastoma biopsies, a gene profiling analysis revealed that *FABP7* expression is inversely correlated to survival in younger patients [16]. Mita et al. [17] showed that in glioblastoma cells FABP7 expression was associated with increased migration. Also Liang et al. [18] and Kaloshi et al. [19] reported that nuclear FABP7 may be induced by EGFR activation to promote migration of GBM tumor cells. FABP7 and EGFR over-expression correlated with short survival in EGFR-positive GBM patients.

These reports point to the relevance of studies on FABP7 influence in gliomas, the subject of this manuscript.

## Materials and Methods

### Primary Cell Line Preparation

Specimens from primary and recurrent glioblastoma tumors obtained from IRCCS Besta (Milan, Italy) under patients informed consent approved by the Institutional board of Ethical Committee of IRCCS Besta, were processed for primary cell line preparation. Tissues were mechanically dissected, trypsin digested at 37°C, to give a monocellular suspension followed by plating the cells in DMEM (GIBCO-Life Technologies, Carlsbad, California/USA #41965-039)/F12 (GIBCO-Life Technologies, Carlsbad, California/USA #21765-029) (1∶1), B-27 (1∶50, GIBCO-Life Technologies, Carlsbad, California, USA), penicillin/streptomycin (1∶100, EuroClone-Milan, Italy), 5 mg/ml heparin, epidermal growth factor (EGF; 20 ng/mL; Tebu-bio, Milan, Italy) and human recombinant fibroblast growth factor 2 (bFGF; 20 ng/mL; Tebu-bio, Milan, Italy). Cells under these culture conditions grow in suspension as cellular aggregates similar to neurospheres of normal neural stem cells. Part of the tumor cells was cultured in medium containing RPMI (Invitrogen #31870-025), 10% heat-inactivated foetal bovine serum (FBS Biowhittaker DE 14-801F). These cells, further mentioned as adherent cells, grew as a monolayer. Over time, the EGF/FGF growing cells form neurospheres and the cells cultured in the presence of serum become adherent but with different range of time. The adherent cells usually are faster than neurospheres cells, taking around three days to get 90% of confluency; neurospheres indeed need three passages (around three weeks, one passage/a week) to form [20].

All the experiments were conducted with cells at passages 19–20.

The cell systems used for this study were reported in Table 1.

**Table 1:** A list of the cells system used in the analysis.

Patient number	Tumor type	Neurospheres	Adherent cells
R11	Recurrent GBM	GBMR11 NS*	GBMR11 AC**
R16	Recurrent GBM	GBMR16 NS	GBMR16 AC
R1	Recurrent GBM	BTR1 NS	BTR1 AC
DBTRG-05MG	Recurrent GBM from Interlab Cell LineCollection (ICLC, Genova, Italy)	DBTRG NS	DBTRG AC
150	Primary GBM	BT150 NS	BT150 AC
165	Primary GBM	BT165 NS	BT165 AC
140	Primary GBM	BT140 NS	BT140 AC
31	Primary GBM	BT31 NS	BT31 AC
138	Primary GBM	BT138 NS	BT138 AC

* NS = Neurospheres.

** AC = Adherent cells.

### Microarray Analysis

For microarray analysis, total RNA was extracted in duplicate from 6 neurospheres and 6 adherent cell lines, further processed, and hybridised to Human Genome U133 Plus 2.0 Array using an Affymetrix GeneChip Instrument System following manufacturer’s recommendations (http://www.affymetrix.com/support/downloads/manuals). Affymetrix CEL files were analyzed with R program (The R Project for Statistical Computing - http://www.r-project.org/) by making use of Bioconductor packages [21]. The analysis was divided into two steps, namely pre-processing and differential expression analysis.

Data pre-processing were considered essential to assign correct expression values to each probe-set and to make different arrays comparable. It consists of background adjustment, normalization, and probe-set expression summarisation. Background adjustment was performed with Robust Multiarray Averaging (RMA) [22] allowing Perfect Match (PM) probes intensities to be corrected by using a global model for the distribution of probe intensities. Normalization for quantitative comparison of different microarrays was performed with quantiles [23] in order to give each chip the same empirical distribution. Final expression values were calculated by summarizing probe-set intensities with Median Polish Algorithm. Transcripts with absolute fold change less than 1.5 were filtered out, and a permutation t-test was applied for determining differential expression. Transcripts with a p value <0.01 were selected for further investigation using Ingenuity Pathway Analysis software, a pathway analysis tool, to better comprehend the biological context of the identified targets.

### Quantitative Real Time RT-PCR

RNA was extracted from cultured cells using the RNeasy mini kit (Qiagen, Milan, Italy). High capacity iScript cDNA synthesis kit (Bio-Rad Laboratories Hercules, CA) was used to reverse-transcribe total RNA (1 µg) in a 20 µl reaction mixture using random primers. Real-time PCR analysis of FABP7 expression in various glioblastoma cells lines was done with the iCycler iQ Real-time PCR using iQ SYBR Green Supermix detection System (Bio-Rad Laboratories, Hercules, CA) and 10 ng of total RNA with the following program: 95°C for 3 sec. followed by 40 cycles of 95°C for 10 sec., 60°C for 30 sec. Each sample was run in triplicate. Hs_FABP7_1_SG QuantiTect Primer Assay (catalog n.QT00007322 QIAGEN) was chosen and used in the reaction mix.

The *FABP7* relative mRNA expression level was calculated using the ΔCt method and normalized with respect to the *GAPDH*, housekeeping gene, which had stable transcript levels under both experimental conditions (adherent and neurospheres).

As controls, normal human astrocytes (NHA LONZA, Basel, Switzerland #CC-2565) and normal human neural progenitors (NHNP LONZA, Basel, Switzerland #PT-2599) were used.

### Western Blot Analysis

The rabbit polyclonal antibody against FABP7 (cat. N. Ab27171) and the rabbit polyclonal antibody against Alpha-tubulin (cat. N. LF-PA0145) and GAPDH (cat. N. G8795) were purchased from Abcam (Cambridge Science Park, Cambridge,UK), from Histo-line (Histo line, Milan, Italy) and from Sigma Aldrich, St Louis, Missouri, USA), respectively.

An aliquot of total protein was separated by 4–20% SDS-polyacrylamide gel electrophoresis and blotted to polyvinylidene difluoride membrane (Invitrogen).

Detection was performed using enhanced chemiluminescence reagent (GE Healthcare) according to the manufacture’s protocol.

### Immunofluorescence Staining

GBMR11 NS and BT150 NS, plated overnight on glass slides coated with fibronectin, were washed once with PBS and then fixed with 4% paraformaldeide for 15 min. After three 5-min gently washes, the cells were permeabilized with 0.1% Triton X-100 for 10 min. After three 5-min washes with TBS (Biorad), the cells were blocked with 3% BSA +1% normal goat serum (Invitrogen) for 30 min. Cells were incubated overnight at 4°C with polyclonal rabbit anti-BLBP (ab32423, 1∶250, Abcam). The next day, cells were carefully washed three times with TBS making attention to avoid the detachment of spheres, and were incubated for 1 h with Alexa Fluor 546-conjugated goat anti-rabbit IgG (Invitrogen). After a subsequent wash with PBS, coverslips were mounted using Prolong Gold antifade reagent with DAPI (Molecular Probes). The staining was evaluated using a fluorescence microscope (Axiovert 200 Inverted, Carl Zeiss, Germany) equipped with a chilled CCD camera (Quantix, Photometrics, USA) and MetaMorph software (Universal Imaging, USA).

### FABP7 Silencing

Cells were passaged 2–3 days before nucleofection with a subcultivation ratio of 1∶2 for BT150 and 1∶4 for GBMR11. Neurospheres were manually dissociated by repeated pipetting up and down to obtain a monocellular suspension. Four million cells for each sample were used and resuspended with 150 µl of nucleofector solution and combined with 100–250 nM siRNA targeting FABP7 (OligoID: Hs_FABP7_6 2024292 Catalog # SI04304286, Qiagen) or negative control AllStars Neg. siRNA Fluorescein (Catalog #1027282,Qiagen) siRNA using nucleofector transfection with the AMAXA system (Lonza). Cells were transfered to an Amaxa certified cuvette into the Nucleofector (LONZA) and were nucleofected with program A-33.

Transfection conditions and times of the two cell lines were optimized using siRNA GFP.

At different time points neurospheres were manually dissociated by pipetting up and down and cells were analyzed by fluorescence microscopy. Transfection conditions and times of the two cell lines were optimized until more than 60% of the cells were successfully transfected with the GFP siRNA. The effect of gene silencing on mRNA levels was evaluated after 48 and 72 hours and the effect on protein levels at 72 and 96 hours after siRNA transfection.

To obtain shFABP7 cells for in vivo study, BT165 NS were transduced with lentiviral particles containing the shRNA sequences using MISSION shRNA Lentiviral Vectors (Sigma Aldrich, St Louis, Missouri, USA), according to the manufacturer’s recommendations. As negative control we used shRNA Lentiviral Particles encoding non specific shRNA (Scrambled cells) or the empty vector (Empty cells). Four days after infection cells were selected for puromycin resistance (1 µg/ml) for one week. A total of 30 nude mice were injected with 1×10^5^ Scrambled or shFABP7 NS (15 mice/group). After injection into the mouse brain of scrambled and shFABP7 cells large gliomas developed and became lethal in two months.

### Proliferation and Migration Assay

The Cell Proliferation Reagent WST-1 (Roche Applied Science, USA) was used to assay NS proliferation and was performed plating 5000 cells/well, as suggested by the manufacturer. The absorbance was evaluated at 450 nm 24, 48 and 72 hours after cell plating. Eight replicates per point were performed. In vitro chemotaxis was assayed using the HTS Transwell-96 system from Corning Inc. (Corning, NY, USA). Neurospheres were manually dissociated by repeated pipetting up and down to obtain a monocellular suspension. A 100 µl portion of cells diluted at 75×10^4^/ml in migration buffer (DMEM with 5% BSA) was placed in the upper wells, whereas the complete medium with growth factors was added to the lower wells. Polyester membranes with a pore size of 1.3 µm were used and incubation was performed at 37°C in a 5% CO2 atmosphere for 48 h. At the end of incubation, migrated cells were detached by placing transwell chambers for 15 min on ice, stained with CyQuant dye and counted using a fluorescence plate reader (M1000 Infinite®; Tecan). All experiments were performed in triplicate.

In order to demonstrate that the observed reduction of growth and migration of tumor cells (in vitro) transfected with *FABP7* siRNA is specifically due to down-regulation of *FABP7*, and not to a non specific (off-target) effect on cell function, we tested the effect of down-regulation of *FABP7* using two other non-redundant *FABP7* siRNAs.

BT150 NS were transfected with 250 nM siRNAs, the first one targeting the *FABP7* 3′UTR (OligoID: Hs_FABP7_2 Catalog #SI00382564,Qiagen;) and the second one targeting the *FABP7* coding sequence (OligoID: Hs_FABP7_7 Catalog #SI04326623, Qiagen) and negative control AllStars Neg. siRNA Fluorescein (Catalog #1027282,Qiagen) using nucleofector transfection with the AMAXA system (Lonza).

After 48 h the silencing efficiency of *FABP7* siRNA was confirmed at the mRNA level (Taqman real-time PCR) and after 72 h at the protein level (by Western blot).

### Irradiation Assays

Two glioblastoma cell lines derived from newly diagnosed tumors, BT150 NS and BT165 NS, were irradiated at 2 Gy using a Faxitron X-ray machine. After 24 and 48 hours the effect of irradiation on FABP7 protein expression and cell migration was evaluated.

### DNA Microarray of BT150, BT165 and GBMR11

For the microarray analysis, total RNA was extracted in triplicate from BT150 NS, BT165 NS and GBMR11 NS, processed and hybridised to Human HGU133 A2.0 Array using an Affymetrix Gene Chip. In the analysis, the common Baseline experimental design has been selected. Once the experimental design has been chosen, the choice of the method depends on the number of available replicates. Limma method was used for the selection of differentially expressed genes (DEG) [24]. Limma is an acronym for Linear Models for Microarray Data (part of the following description is drawn from the Limma vignette). It is a Bioconductor library developed by Gordon Smyth [25]. This method is based on the fitting of a linear model to estimate the variability in the data. In case of one-channel microarray data (like Affymetrix) this approach is the same as analysis of variance except that a model is fitted for every gene. For the detection of the differential expression an empirical Bayes method is used to moderate the standard errors [25]. Indeed the use of moderated statistics for the detection of differential expression is very useful especially in cases of experiments with a small number of replicates. In this analysis, all the requested comparisons were performed selecting DEG with a threshold pValue of 5e-04 and FoldChange cutoff equal to 1.5.

In order to establish gene profile on BT150 NS, BT165 NS and on GBMR11 NS, we compared expression of selected proneural (PN), mesenchymal (Mes) and classical/proliferative (Prolif) markers [26], [27].

### In Vivo Experiment and Immunohistochemistry Analysis

BT165 NS were injected orthotopically into the left striatum of mouse brain (CD-1 nu/nu, Charles Rivers, Calco, Italy). All mice were maintained in a conventional-specific-pathogen-free facility according to the NIH guidelines using an approved Animal Care and Use Committee protocol. The study was conducted in compliance with Decreto Legislativo January 27, 1992, N. 116, Gazzetta Ufficiale N. 40 February 18, 1992, (Directive N. 86/309/CEE) concerning protection of animals used for scientific purposes (project authorization No. 48/2009/B).

Mice were placed on the stereotaxic apparatus with the head placed in the anesthesia cone (inhalant anesthesia with isoflurane) and gently fixed with ear bars and into the head holder. Skin of the head was cut longitudinally and skull exposed. After defining Bregma as Y = 0 and X = 0 in the apparatus, the point of injection was identified as +0.5 mm anterior and +2.2 mm lateral (right). A small hole was done with a micro drill and a Hamilton syringe, loaded with 5×10^5^ tumor cells in 5 µl of PBS 1X just prior injection, was gently inserted into the brain until reaching −3.0 mm depth. Cells were injected at the speed of 0.5 µl/min. The syringe was left in place for additional 5 min to avoid cell aspiration. Hole was closed using bone wax and the wound sutured with sterile autoclips. After the end of the surgery mice were monitored for recovery until complete awakening. Mice were examined regularly for the appearance of clinical signs of tumor growth.

Brains were collected after 55 days for immunohistochemistry analysis. Paraffin embedded sections were analyzed with the following antibodies: polyclonal rabbit anti-BLBP (Abcam), anti-Ki67 (BD-Biosciences, Franklin Lakes, New York, USA) and anti-doublecortin (DCX, Abcam).

The absolute cell number of positive cells was calculated in 8 non overlapping high power fields. Results have been expressed as mean number of positive cells ± SD for each group. The positive rates were counted thrice manually from the photographs by two observes. Student’s T-test was performed for evaluating the significance of data. Statistical significance was determined at the = or <0.05 level.

## Results

### FABP7 is Highly Expressed in Glioblastoma Neurospheres

**Figure 1:**
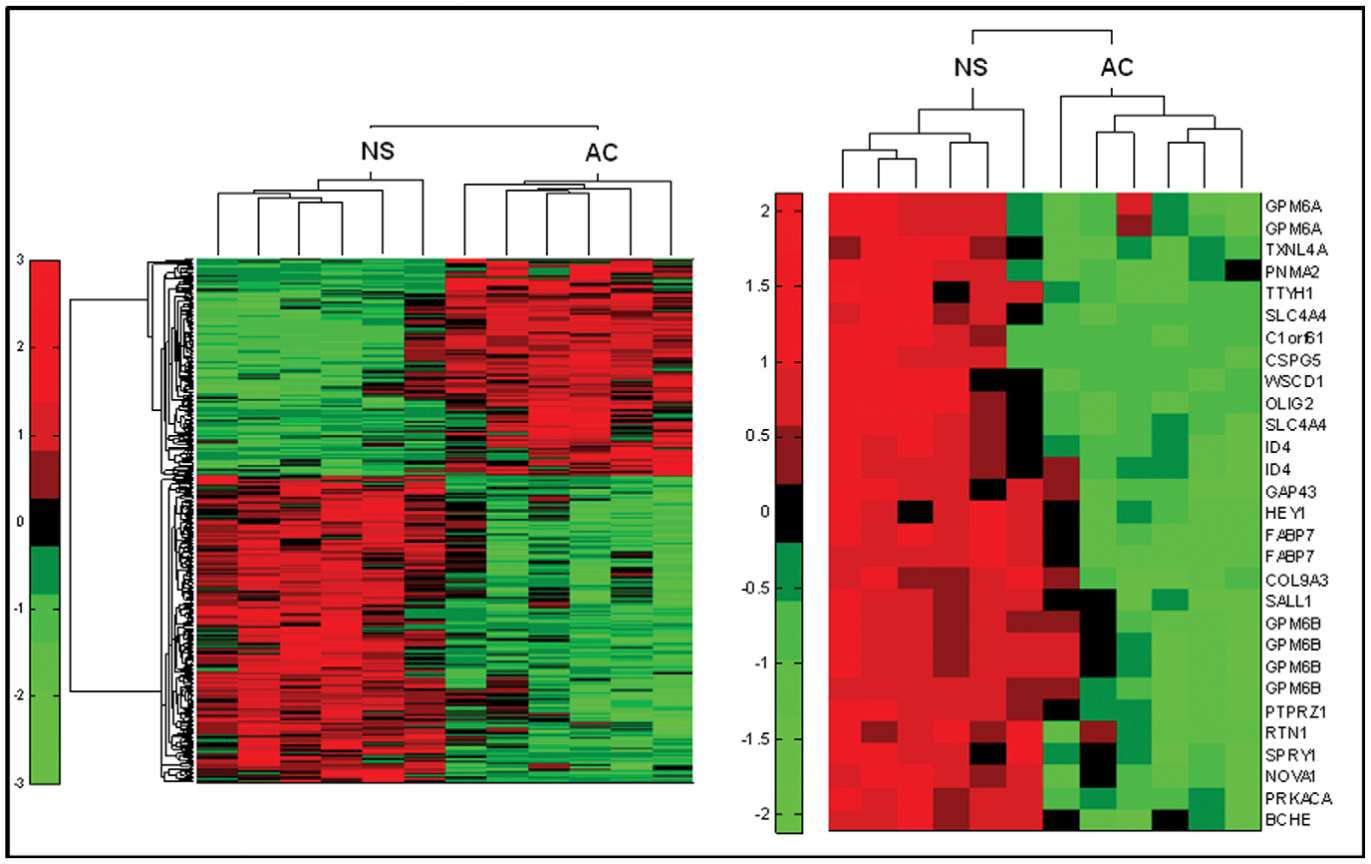
Microarray analysis of glioblastoma adherent cells versus neurospheres. The microarray experiment was done in duplicate on 6 neurospheres and 6 adherent cells. Hierarchical clustering of 617 transcripts using Pearson correlation (between transcripts) and Euclidean distance (between samples). The right panel of the figure shows the 29 most up regulated transcripts (fold change higher than 10) corresponding to 22 different transcripts.

A total of 617 transcripts were selected as differentially expressed in GBM neurospheres or adherent cells with absolute fold change >1.5 and permutation t-test p value <0.01. The left panel of Figure 1 shows a bi-dimensional cluster analysis based on row standardized values using Pearson correlation as distance metric between rows (transcripts) and Euclidean distance between columns (samples). The right panel shows the 29 most up regulated transcripts (fold change >10) corresponding to 22 different transcripts. Neurospheres (NS) compared with adherent cells (AC) show increased expression of stemness genes such as *OLIG2, ID4*, and *HEY1. FABP7* resulted one of the most up-regulated transcripts (Figure 1).

**Figure 2:**
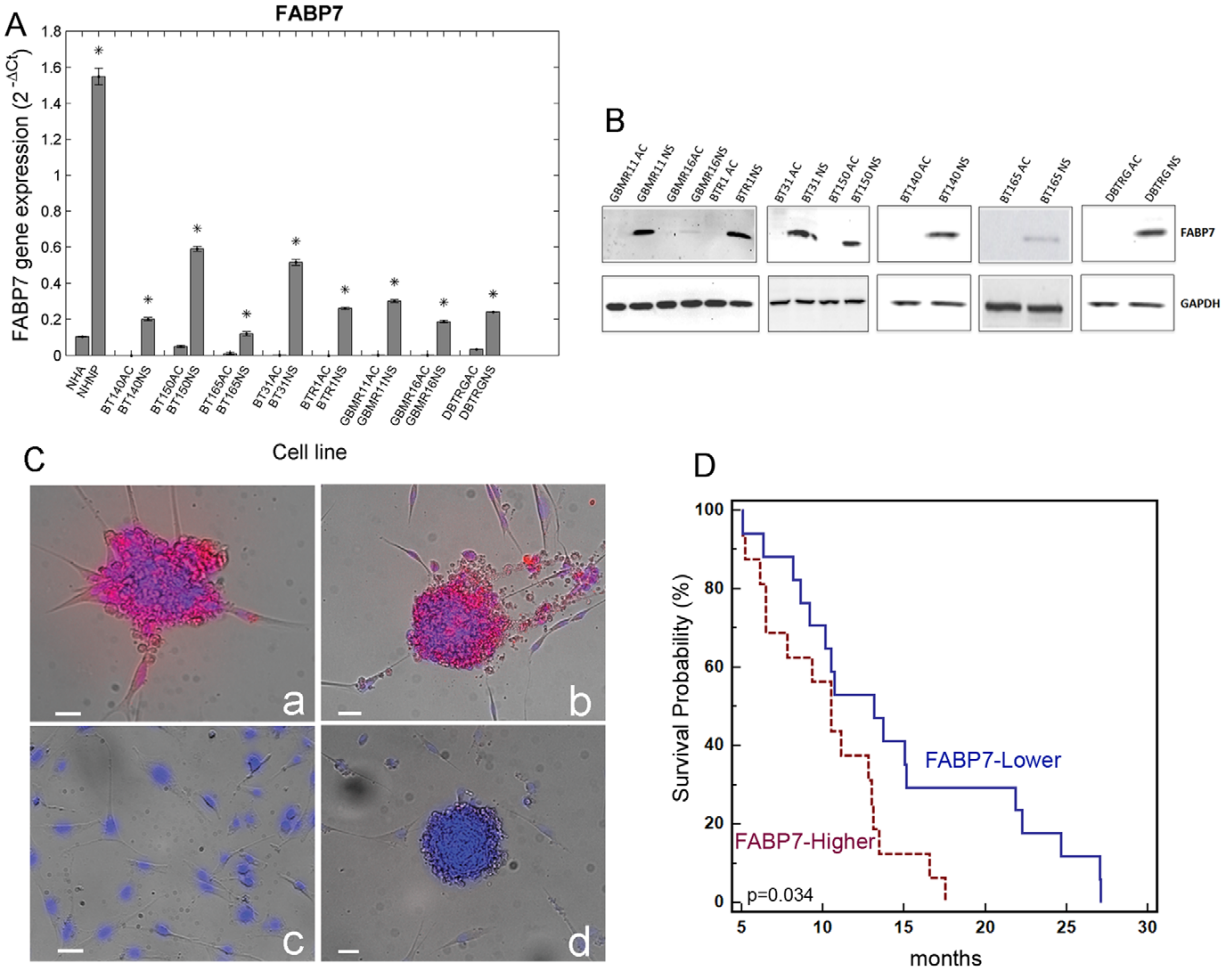
FABP7 expression in various glioblastoma cell lines. (A) *FABP7* mRNA in various glioblastoma cell lines. Quantitative real time PCR of primary and recurrent glioblastoma show a significantly higher expression of *FABP7* in the neurospheres compared to the adherent cells derived from the same patient’s tumor. Normal Human Astrocytes and Normal Human Neural Progenitors were used as controls. *p<0.05. (B) Expression of the FABP7 protein in glioblastoma with Western blot analysis. In lysates from neurosphere cell lines of primary and recurrent glioblastoma patients a 15 kDa FABP7 band was detected. No band was observed in lysates from their adherent counterpart. GAPDH protein was used as an internal control. (C) Immunofluorescence analysis of FABP7 protein expression in glioblastoma cell lines. While the cytoplasm of the neurospheres (a and b) stained positive for FABP7 the adherent cells (c) were negative. Negative control (secondary antibody only) is represented in panel (d). Scale bar = 20 µm. (D) Kaplan Meier survival analysis. The overall survival of GBM patients expressing lower levels of FABP7 (n = 17) survived longer than patients with a higher expression of FABP7 (n = 16).

In order to validate the observations from the microarray experiments, the level of *FABP7* mRNA in different cells from primary and recurrent glioblastoma patients was measured using quantitative real time PCR. Primary and recurrent glioblastoma neurospheres were positive for *FABP7* expression whereas in the adherent cells no (Ct ≥40) or significantly lower *FABP7* expression was observed: normal human astrocytes and normal human neural progenitors were used as controls (Figure 2A). The fold change values are reported in Table 2.

**Table 2:** Fold change values of quantitative real time PCR of neurospheres (NS) versus adherent (AC) glioblastoma cells.

Cell Line	NS vs AC
NHNP vs NHA	14.93
BT140	NA
BT150	11.80
BT165	12.00
BT31	2410.12
BTR1	NA
GBMR11	3512.07
GBMR16	160.08
DBTRG	7.16

To evaluate the expression of FABP7 at the protein level, Western blot analysis was carried out: as shown in Figure 2B, the 15 kDa band corresponding to FABP7 was detected in all glioblastoma neurospheres, independent of whether they derived from primary, recurrent tumors, or from a commercially available glioblastoma cell line (DBTRG). The same band was absent in adherent cells derived from the corresponding patients.

As depicted in Figure 2C, also immunofluorescence staining confirmed the Western blot observations.

We also studied the contribution of FABP7 expression to patient overall survival (OS). We analyzed mRNA expression levels in 33 GBM specimens from patients treated with standard chemo-radiotherapy (mean 2^−ΔΔct^ ± SD: 6.6±6.2 vs normal brain p<0.0001; median: 6.1). We subdivided two groups of GBM identified by higher (FABP7^High^, 2^−ΔΔct^ >6.1, n = 16) and lower (FABP7^Low^, 2^−ΔΔct^ <6.1, n = 17) levels of FABP7 expression (mean ± SD: 10.2±6.6 and 3.1±2.3, respectively, p = 0.0004). FABP7^Low^ patients had significantly longer OS than FABP7^High^ patients (median OS 13.2 vs 10.5 respectively, p = 0.034, Figure 2D).

### FABP7 Expression and Cell Culture Conditions

**Figure 3:**
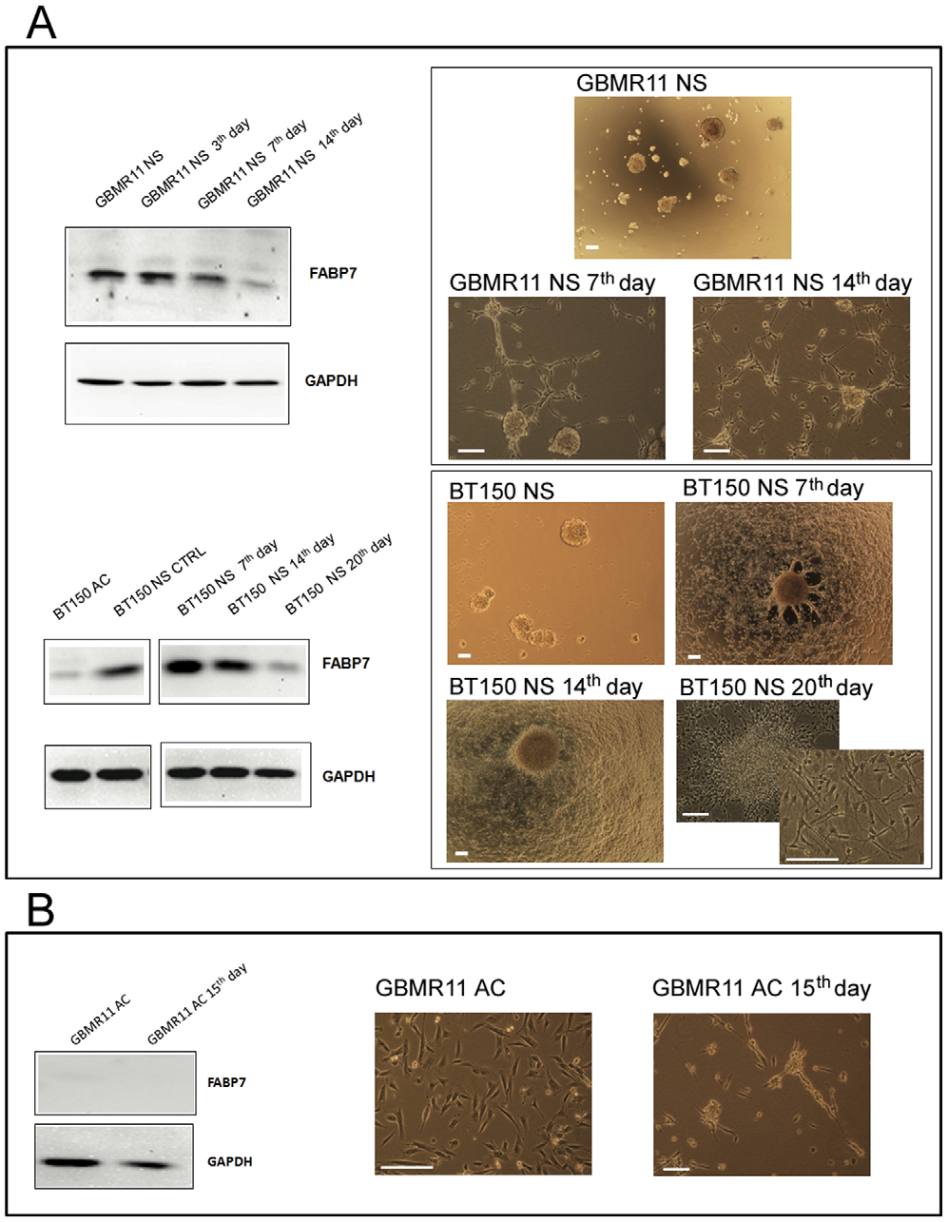
Impact of cell culture conditions on FABP7 protein expression. (A) Seven days after withdrawal of growth factors and addition of serum, FABP7 expression decreased together with the change in morphology from neurospheres to adherent cells. (B) In adherent cells cultured with growth factors, no FABP7 expression was observed. Scale bar = 200 µm.

To exclude that FABP7 increased expression in neurospheres versus adherent cells is caused by culture conditions rather than by intrinsic properties of GBM cells growing as NS, the expression of FABP7 was assessed by culturing neurospheres in growth factors free medium composed by RPMI plus 10% FBS and evaluated at different time points. After 7 days in serum containing medium, morphological changes were observed in a low number of neurospheres, but overall FABP7 expression remained stable in the total cell population. A significant drop in FABP7 expression was observed after 14 days for GBMR11 NS and 20 days for BT150 NS. The latter time point coincided also with a change in cellular morphology, as the majority of cells become adherent (Figure 3A). No FABP7 expression was observed in adherent cells upon exposure to DMEM/F12 plus growth factors medium (Figure 3B): 15 days after the addition of this medium to the cells, no evidence of FABP7 expression was found. Thus, the presence of serum in the medium, by inducing the differentiation, correlates with absent/decreased of FABP7 expression. In agreement with this hypothesis, normal human neural progenitors cells have significantly higher FABP7 expression compared to normal human astrocytes (Figure 2A).

### FABP7 is Involved in Migration and Cell Proliferation of Glioblastoma Cell Lines

To investigate the functional role of *FABP7*, we examined the effects of *FABP7* down-regulation with specific siRNA on the invasive and proliferative capacity of glioblastoma derived neurospheres: BT150 NS and GBMR11 NS, derived from a newly diagnosed and a recurrent GBM, respectively.

BT150 NS and GBMR11 NS were transfected with *FABP7* and with control siRNA. After 72 h silencing efficiency of *FABP7* siRNA was confirmed by real-time PCR and Western blot in both cell lines.

**Figure 4:**
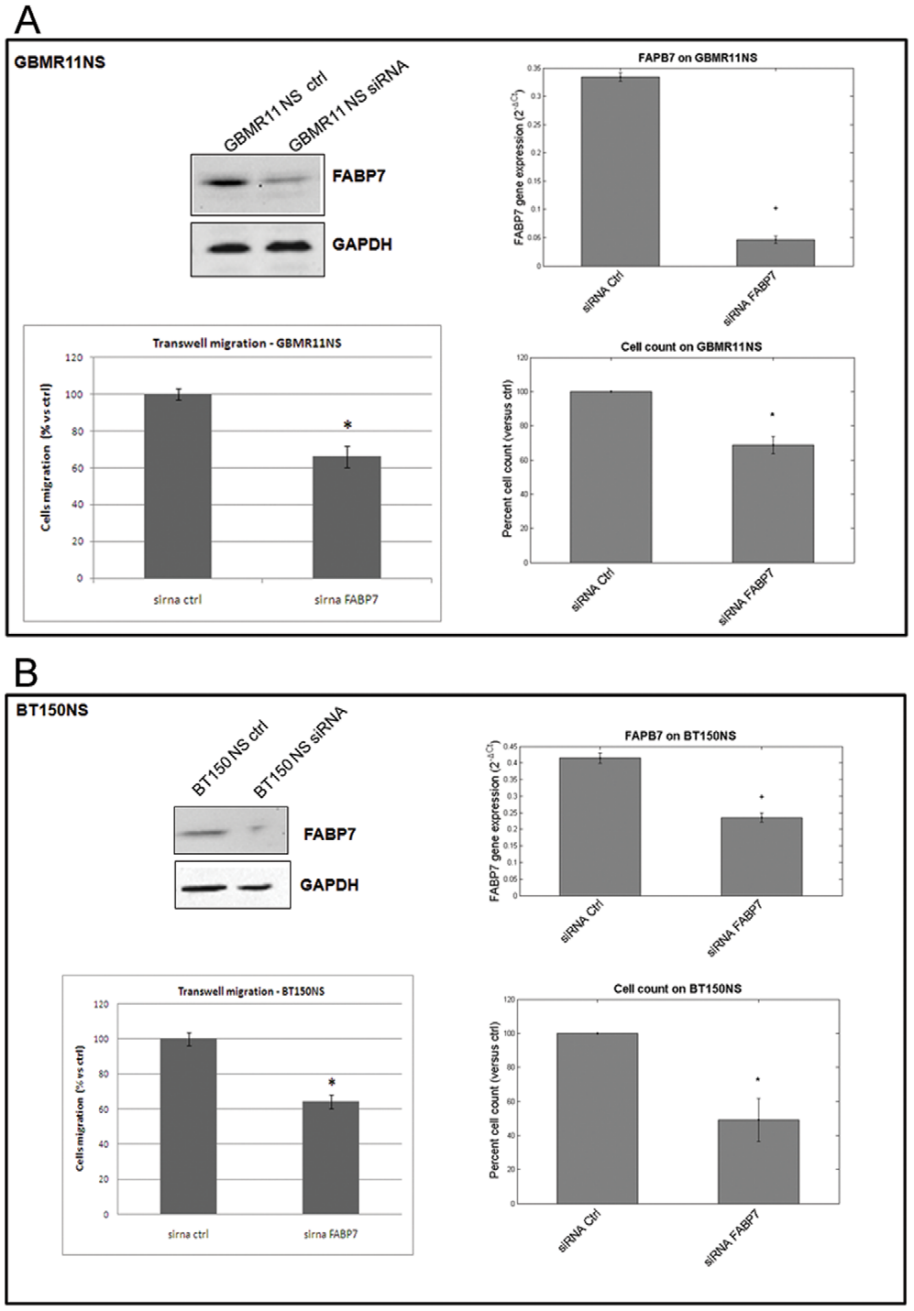
FABP7 silencing affects glioblastoma cell migration and proliferation. Transfection with si*FABP7* in neurospheres resulted in a significant reduction of both proliferation and migration independent of whether the cells were derived from recurrent (GBMR11, panel A) or primary (BT150, panel B) glioblastoma. *p<0.05.

As demonstrated in Figure 4, FABP7 down regulation reduced proliferation by 30% (±5 SEM) in GBMR11 and by 50% (±12 SEM) in BT150 NS as compared to scrambled siRNA controls (Figures 4A and 4B, respectively).

We also investigated the effect of FABP7 down regulation on migration using the transwell system. The number of migrating cells after transfection with FABP7 siRNA compared with scrambled siRNA control-transfected cells was significantly reduced by 44% and 46% in GBMR11 and BT150 NS, respectively (p<0.05; Figures 4A and 4B). These results suggest that FABP7 contributes to the proliferation and motility of glioblastoma cells. To further confirm these results, we also tested other FABP7 siRNA and observed a reduction of cellular migration and proliferation associated to FABP7 down-regulation. As depicted in Figure S1, the silencing capacity of siRNA_2 and siRNA_7 (both at mRNA and protein level) is less prominent than the previously used siRNA (see Figure 4). The effect of *FABP7* downregulation was then analyzed in proliferation and migration assay. The phenotypic effect of these siRNAs is in line with their silencing capacity.

### Irradiation of Glioblastoma Neurospheres Enhances FABP7 Expression, Cell Migration and Cell Proliferation

For the irradiation experiments we selected two GBM-neurosphere cell lines with different FABP7 expression: BT150 NS with high expression and BT165 NS with lower expression.

The effect of irradiation on FABP7 expression was assessed by exposing the neurospheres to 2 Gy and quantifying FABP7 protein content after 24 and 48 h using western blot analysis.

In order to appraise the effects of irradiation and the consequent increased FABP7 expression on the migration ability of the GBM NS we performed the transwell migration assay.

**Figure 5:**
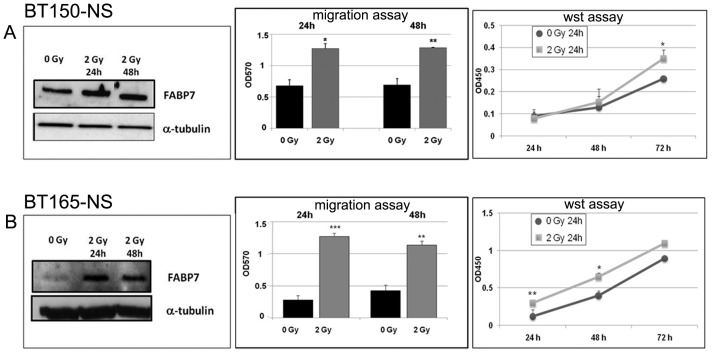
Irradiation enhances FABP7 expression and glioblastoma cell migration and proliferation. Irradiation at 2 Gy resulted in a clear increase in FABP7 protein expression after both 24 and 48 h in BT150 NS (A) and BT165 NS (B). 15 µg for BT150 NS and 30 µg for BT165 NS of proteins were loaded in each well. Loading was normalized for α-tubulin. Significant difference in migration was observed at the same time points in BT150 and BT165 (*p<0.05). Results derived from two independent experiments. Proliferation analysis, evaluated at 24, 48 and 72 hours, indicates that after 2 Gy irradiation BT150 NS proliferate significantly more than after 0 Gy, only 72 hours after plating, while 2 Gy-irradiated BT165 NS proliferate significantly more than 0 Gy-irradiated cells, 24 and 48 hours after plating (*p<0.05; **p<0.001).

The migration assay was carried out for 24 and 48 h after irradiation (Figure 5). For both cell systems and at both time points, 2 Gy irradiation resulted in a significant increase of cell migration and FABP7 expression. As depicted in Figure 5, 24 as well as 48 hours after 2 Gy irradiation BT150 NS cells showed a similar increase in FABP7 expression and migration (1.9 folds vs 0 Gy). For BT165 NS cells after 24 h, 2 Gy irradiation induced FABP7 expression more importantly and migration was increased by 4.5 fold vs 0 Gy; 48 h after irradiation, both FABP7 protein expression and migration decreased (2.6 fold vs 0 Gy).

We further evaluated the effects of irradiation and increased FABP7 expression on proliferation of both NS cell lines. Proliferation assay performed 24 h after 2 Gy irradiation and evaluated at three different time points (24 h, 48 h, 72 h) showed that irradiated BT150 NS proliferate significantly more than control (0 Gy) 72 h after plating cells (p = 0.04). For BT165 NS cells 2 Gy irradiation induced a more significant increase in proliferation compared to control (0 Gy) 24 h and 48 h after plating cells (24 h p<0.001; 48 h p = 0.01).

### Functional Role of FABP7 in vivo

**Figure 6:**
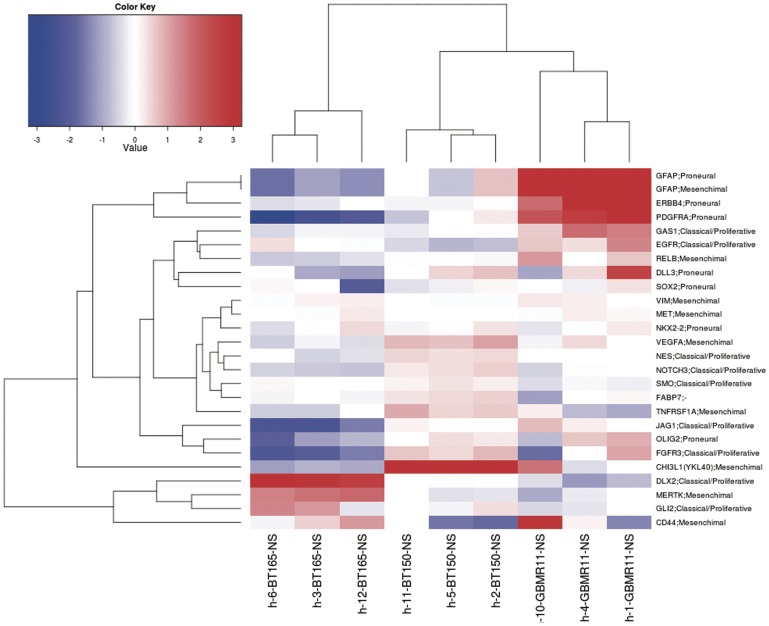
Summary of selected proneural, mesenchymal and classical/proliferative markers up- or down- regulated in BT150 NS, BT165 NS and GBMR11 NS. The heat map represents a summary of selected markers in order to predict mesenchymal (Mes), proneural (PN) and classical/proliferative (Prolif) subclasses. Several Mes genes are identified upregulated in BT150 NS such as *YKL40* (*CHI3L1)*, *Vimentin*, *TNFRS1A*, *VEGFA* and *GFAP* (marker more strongly expressed in Mes and PN subclasses). In addition *OLIG2, PDGFRA, SOX2, DLL3, ERBB4* and *GFAP* (PN markers) are upregulated in GBMR11 NS. However two important features are highly identified and linked to Prolif subclass: the expression of *DLX2,* a marker of transit-amplifying cells and the absence of *GFAP* expression, both findings are present in the BT165 NS microarray profile.

To evaluate the functional role of FABP7 we performed *in vivo* experiments using two cell lines: BT150 NS and BT165 NS. A gene profile analysis was performed on these cell lines (see heat map in Figure 6). We identified genes up-regulated in BT150 NS that supported its inclusion into the Mes subclass. These genes included *YKL40*, absent in BT165 NS, *Vimentin*, *TNFRSF1A*, *VEGFA* and also *GFAP*, marker more strongly expressed in both Mes and PN subclasses and absent in BT165 NS. On the contrary BT165 NS express *DLX2*, an important marker of amplifying cells strongly expressed in Prolif subclass and resulting absent in BT150 NS. All of PN markers are up-regulated in GBMR11 NS (*OLIG2, PDGFRA, SOX2, DLL3, ERBB4* and *GFAP*) suggesting the inclusion of this cell line in the PN GBM subtype.

Moreover, we confirmed the higher expression of FABP7 in BT150 NS than in the BT165 NS (see Figure 2).

BT150 NS and BT165 NS were transduced with lentiviral particles expressing shRNA targeting FABP7 (shFABP7) using as negative control lentiviral particles encoding non specific shRNA (Scrambled). In the BT150 NS cell line we obtained a strong reduction of FABP7, verified by RT-PCR (96% of inhibition vs scrambled), and growth inhibition in vitro. Ten days after plating, shFABP7 NS appeared small and disrupted, when compared to scramble NS, or attached to the plate, showing signs of morphological changes (see Figure S2). This dramatic effect on tumor cell growth did not allow to obtain enough cells to perform in vitro and in vivo experiments testing the effects of FABP7 inhibition.

**Figure 7:**
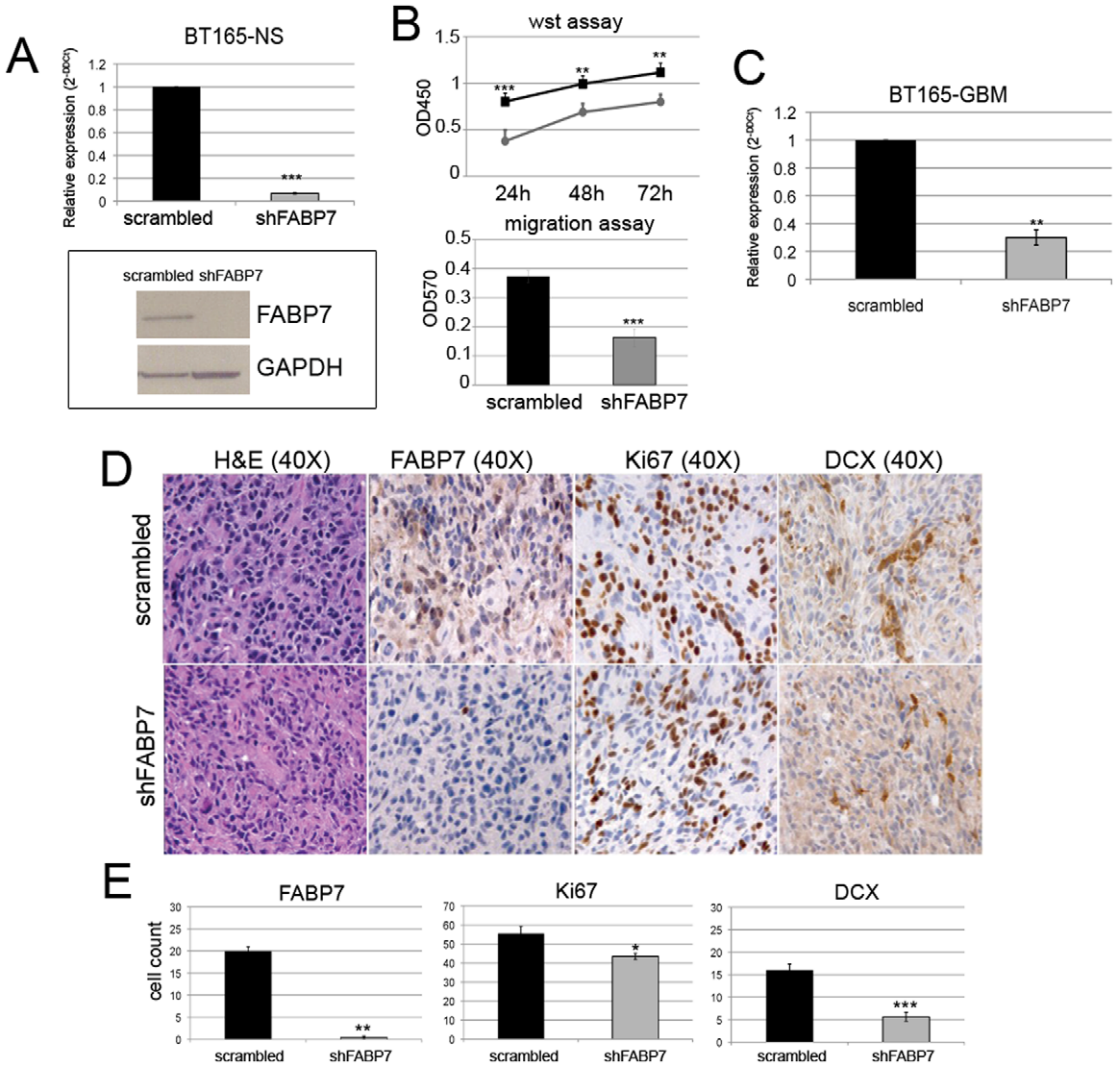
Silencing of FABP7 on BT165NS cell line. Effect of silencing of FABP7 on BT165 in vitro and in vivo. Real time PCR performed on BT165 NS cells after the silencing shows high efficiency of the inhibition (compared to scrambled) (Panel A). Effects of FABP7 inhibition on in vitro proliferation and migration in shFABP7 NS compared to scrambled NS are reported on Panel B. Scrambled infected cells are reported in black and shFABP7 cell in grey (**p<0.02, ***p<0.001, see text for additional details). RT-PCR confirmed that a strong silencing of FABP7 was maintained during in vivo tumor growth (70% of inhibition vs scrambled tumors, Panel C, ***p<0.003). Immunohistochemistry analysis showed that a significantly lower number of FABP7 and Ki67 positive cells was found in shFABP7 tumors as respect to scrambled (Panel D) (*p<0.003 and **p<0.05, respectively). Gliomas deriving from shFABP7 NS showed very low expression also of DCX (***p<0.001). Three representative mice for each group have been investigated and representative images for each tumor are displayed. Histograms reported on Panel E represent the immunohistochemistry quantification of FABP7, Ki67 and DCX positive cells in scrambled and shFABP7 tumors. Quantification was made by counting five different fields for each slide.

In BT165 NS, silencing of FABP7 gene expression was similarly efficient as confirmed by real-time PCR and western blot analysis (Figure 7, panel A). Effects on cell growth, however, were less dramatic, allowing to perform in vitro tests that showed decreased proliferation and invasion in BT165 silenced for FABP7 vs control cells (Figure 7, panel B). Proliferation assays performed at three different time points (24 h, 48 h, 72 h) showed that shFABP7-NS proliferate significantly less than scrambled NS (24 h ***p<0.001; 48 h **p<0.02, 72 h **p<0.02), and have significantly lower migration capacity compared to scrambled cells (2.3 fold vs scrambled cells, ***p = 0.003) (Figure 7, panel B). However, in vivo survival of mice injected with BT165 NS infected cells (scramble and shFABP7) was similar (not shown) in spite of evidence of maintained down-regulation for FABP7 expression ex-vivo, confirmed by FABP7 positive cell counts (***p = 0.003, vs scrambled tumors; Figure 7, panels D and E) and real time PCR (Figure 7, panel C). Doublecortin (DCX) plays a crucial role in neuroblast migration and since it was reported that DCX is preferentially expressed in invasive gliomas [28], [29], the sensitivity and specificity of DCX as a marker for infiltrating glioma cells was immunohistochemically evaluated. Moreover, the intra-cranially growing tumor derived from shFABP7 transduced cells showed a significantly lower DCX expression than in scrambled NS (***p<0.001), suggesting that FABP7 play a role in the invasion rather than in the proliferative process.

Since BT150 NS are less proliferative and more invasive than BT165 NS and based on the differences observed in the two cell types after silencing it can be hypothized that FABP7 play a more relevant functional role in the mesenchymal rather than proliferative subtype of GBM.

### FABP7 Upstream and Downstream Network Analysis

**Figure 8:**
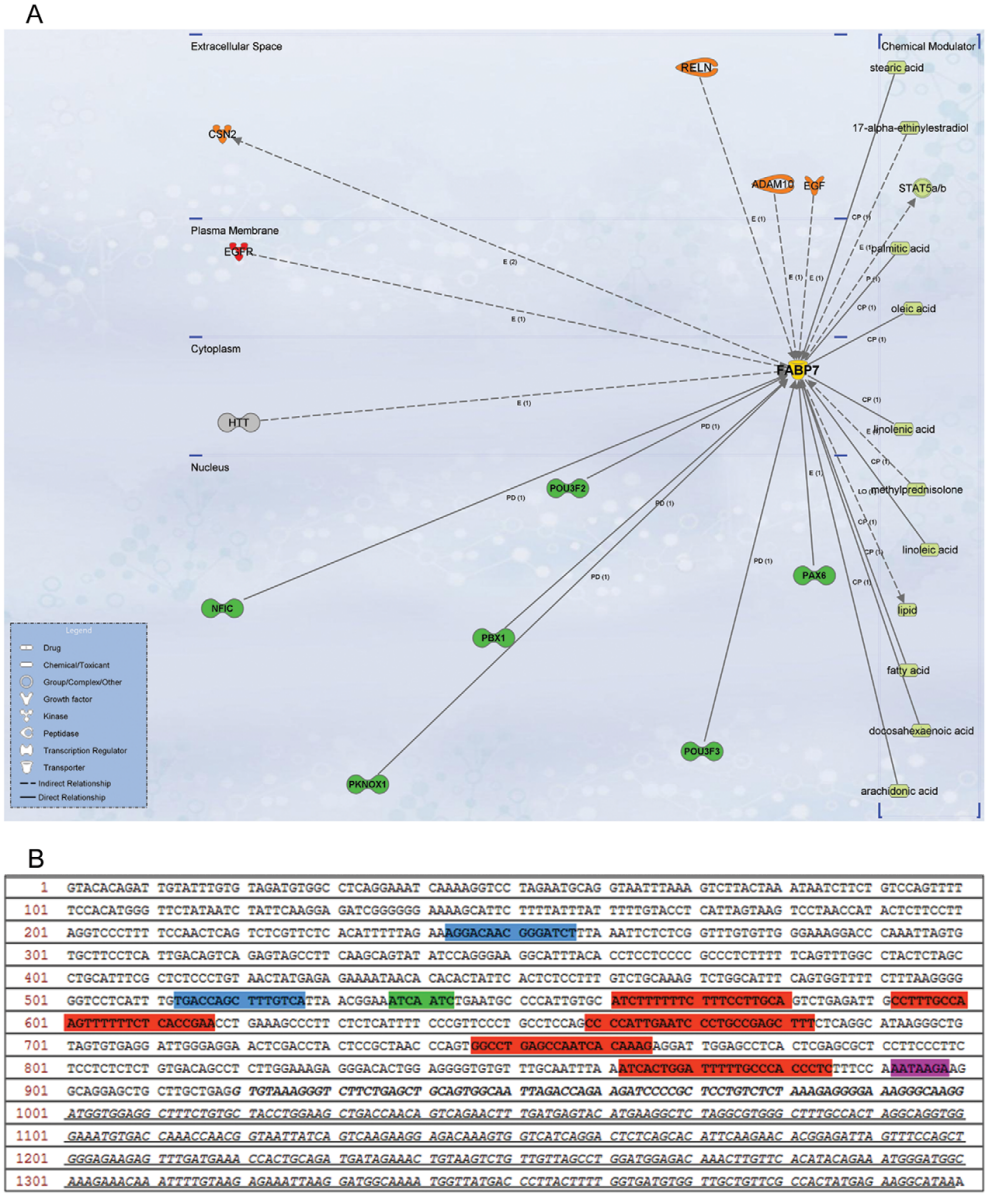
Bioinformatic analysis of FABP7 pathway. (A) Network of *FABP7* interaction partners (Transcription factors, green). (B) 5′ Flanking region of *FABP7* TSS (black italic); TATA box (purple); binding site of NFIC (red) and POUs (green); hypothetical regions of PPRE for PPAR (blue).

A bioinformatics analysis was performed with the objective of drawing an upstream and downstream network of proteins interacting directly and indirectly with FABP7. In order to assess the role of *FABP7* in the context of its gene expression and its protein role as fatty acid carrier a first step was to analyze the interaction network from Ingenuity Pathway Analysis. The neighbourhood network of *FABP7,* as reported in Figure 8A, shows the binding of transcription factors such as POU3F2, POU3F3 (POU family), PBX1, PKNOX1, and PAX6 to the *FABP7* promoter region. A further literature analysis revealed that NFIC has 5 binding sites [30], POU and Pax6 transcription factors one binding site [31]–[32]. Additionally, evidence for binding of PPAR to its responsive element region (PPRE) [33] as well as Coup-TF1 to the *FABP7* promoter region was determined [34].

The first 1000 bp of the *FABP7* 5′ Flanking region (Ensembl ID ENSG00000164434) were analyzed and all DNA motifs belonging to mentioned transcription factors were mapped on this region (Figure 8B). The PPAR responsive element (PPRE) motif (AGGTCA-C-AGGTCA) was mapped on the *FABP7* sequence in order to hypothesize potential binding sites of PPRE to *FABP7*. More likely regions of PPRE binding to FABP7 are indicated in blue (Figure performed with Vector NTI 10, Invitrogen).

### Influence of PPAR Antagonists on FABP7 Gene Expression

**Figure 9:**
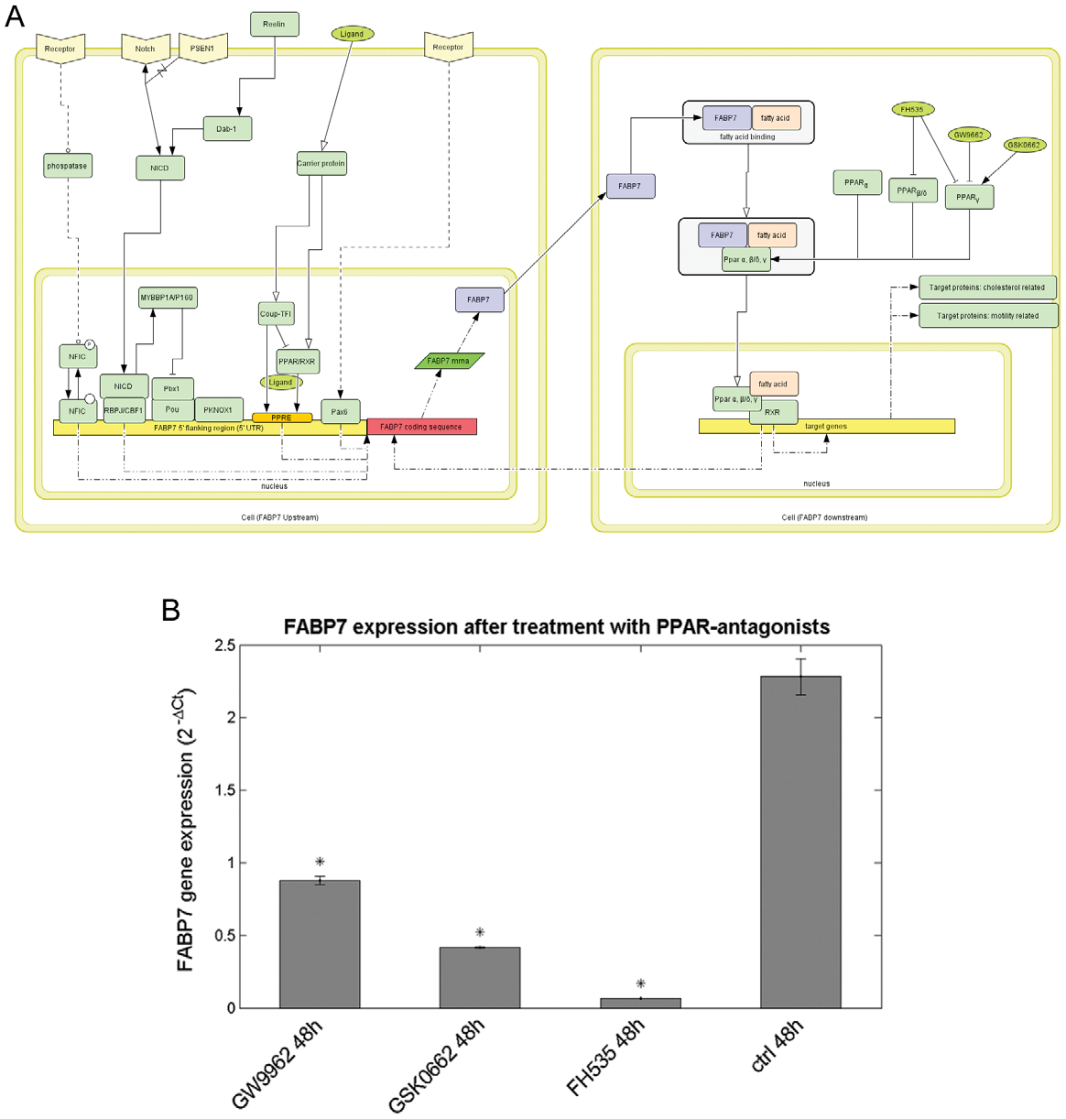
Effect of PPAR antagonists on FABP7 expression. (A) Bioinformatic analysis on FABP7 pathway. Overview of upstream and downstream pathway of FABP7 drawn with Cell Designer 3.5.2 (System Biology Institute). (B) mRNA *FABP7* expression after PPAR antagonists treatment. High *FABP7* expressing GBMR11 neurospheres were treated for 48 h with 15 µM of antagonists against PPAR δ/γ (FH535), PPARγ (GW9662) and PPAR δ (GSK0660). Although *FABP7* mRNA expression is affected by all PPAR antagonists, FH535 is most effective (*p<0.05).

A bioinformatics analysis was performed in order to assess the role of FABP7 (Figure 8). From literature and pathway analysis it appears that PPAR plays a significant role as a key factor modulating the transcriptional activity of FABP7: in agreement with this, a feedback loop between PPAR and FABP7 was identified by our analysis. Peroxisome proliferator–activated receptors (PPARs), members of the nuclear receptor superfamily of transcription factors, are known for their critical role in the development of different diseases, including obesity, cardiovascular disease, type 2 diabetes and cancer. Three PPAR receptor subtypes (PPAR α, β/δ, and γ) have been discovered that differ in tissue distribution, physiologic functions, and ligand specificity. Since our bioinformatics analysis suggested that FABP7 is a PPAR target gene, the effect of known PPAR inhibitors on FABP mRNA expression was verified. GBM neurospheres with high expression of FABP7 were treated for 48 h with three different small molecule PPARs antagonists: FH535 that antagonizes both PPARγ and PPARδ activity, GW9662 which is a selective PPARγ antagonist, and GSK0660 a PPARδ antagonist (all 15 µM). FABP7 expression, measured by qRT-PCR, was affected by all three PPAR antagonists, but the dual δ/γ PPAR antagonist FH535 appeared the most effective one (Figure 9).

We also evaluated the effects of PPAR antagonists by treating BT165-NS for 24 and 48 h after irradiation.

**Figure 10:**
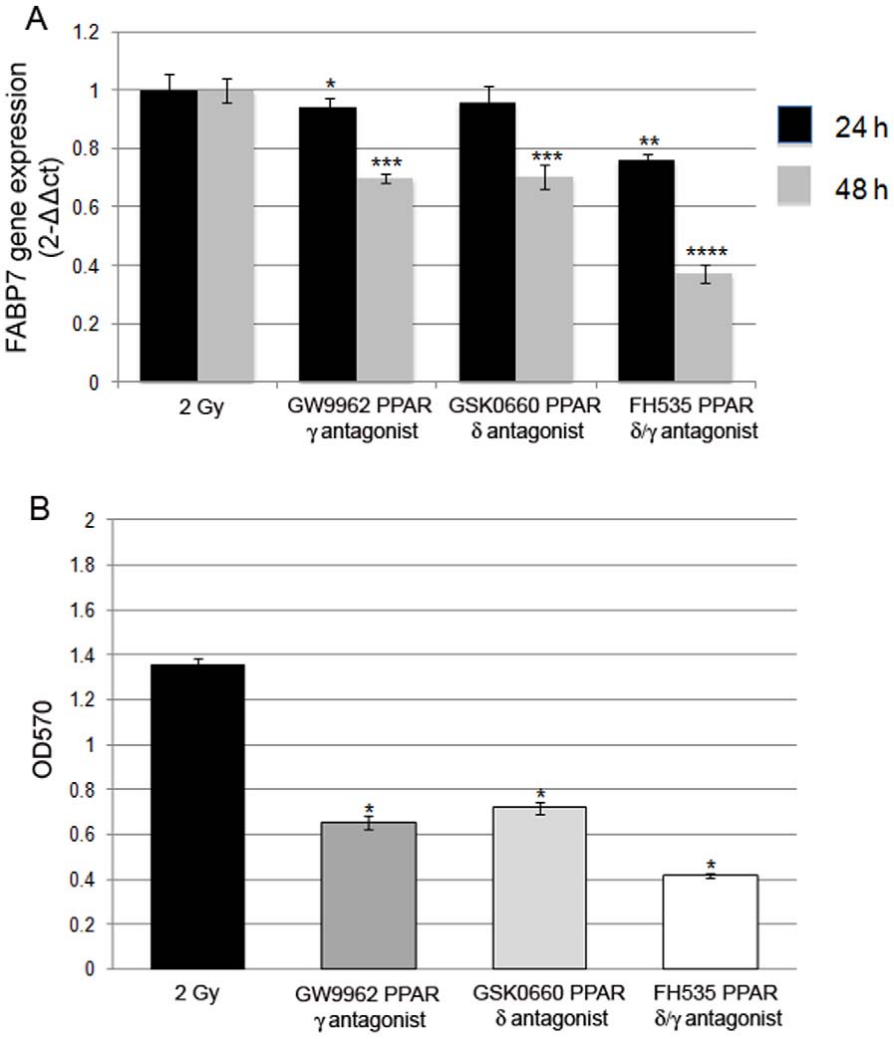
Effect of PPAR antagonists on FABP7 expression in irradiated cells. (A) Real time PCR analysis on irradiated BT165 NS during PPAR antagonists. BT165 NS were irradiated with 2 Gy dosage and treated for 24 and 48 h with 15 µM of antagonists against, PPARγ (GW9662), PPAR δ (GSK0660), PPAR δ/γ (FH535). FABP7 expression was significantly modulated especially after 48 h of treatment (* p<0.05, p<0.001, p<0.0001). (B) Migration assay on irradiated cells after 48 h of antagonist treatments. BT165 NS showed a significant decrease of migration ability compared to irradiated cells (p<0.05).

FABP7 expression was 3.6±0.5 folds higher in NS 24 h after irradiation than unirradiated cells (p<0.001, data not shown). During treatment with PPAR antagonists irradiated NS down-regulated FABP7, and this correlated with a significant decrease of migration ability (Figure 10), confirming the correlation between FABP7 expression and migration.

## Discussion

In the present study, we identified for the first time that *FABP7* is almost exclusively expressed in neurospheres and not in the adherent cells, derived from the same glioblastoma tumor. This observation was confirmed in cells from both newly diagnosed and recurrent tumors [35]. We found that FABP7 was expressed in two long-term established cells lines growing adherently with serum, U251MG and M049 but not in two other such cell lines, U87 and M002. Long-term cell lines growing in serum tend to re-shape the genetic features that were originally present in the tumor specimen [11], [36]. This fits with the observation that serum-cultured GBM cells may or may not conserve FABP7 expression, as found by Godbout et al. [35], while in NS cells from GBM the FABP7 expression pattern of original GBM is more conserved. We also found that FABP7 expression is associated with shorter survival as also reported by Kaloshi et al [19].

In the current study we showed that only GBM cells growing as neurospheres express FABP7.

Real time PCR and Western blot analysis revealed that FABP7 was uniquely expressed in neurospheres and almost absent in adherent cells independent of whether the cells were derived from a primary or a recurrent tumor. Moreover, silencing of FABP7 affected growth, and resulted in a reduced invasiveness, and altered morphology of the neurosphere-derived cells (NS). Interestingly, we also showed that low Gy irradiation resulted in induction of FABP7, leading to increased cell migration and proliferation.

In order to explain the potential connection between FABP7 and increased migration, especially after irradiation, we suggest the following working hypothesis.

Chemotherapy and irradiation may cause oxidative stress and ischemia. Bazan et al [37] showed that in rat brains challenged by oxidative stress (ischemia-reperfusion), the DHA derivative neuroprotectin D1 (NPD1) is synthesized. In addition, when NPD1 is infused during ischemia-reperfusion or added to RPE cells during oxidative stress, apoptotic DNA damage is down-regulated. NPD1 also up-regulates the anti-apoptotic proteins Bcl-2 and Bcl-xL and decreases pro-apoptotic Bax and Bad expression, inhibits oxidative stress-induced caspase-3 activation and IL-1beta-stimulated expression of COX-2. Overall, NPD1 protects cells from oxidative stress-induced apoptosis [37]: therefore, the synthesis of DHA could be a survival response to oxidative stress induced by chemotherapy and irradiation.

Only cells expressing FABP7 will have the capacity to transport molecules such as DHA to the plasma membrane. Once in the membrane DHA may induce changes in membrane fluidics and increase cell plasticity and motility [38]. This may explain why neurospheres have a higher capacity to invade and thereby escape cell killing.

On the contrary, Mita et al. [39] reported that in U87 adherent glioma cells DHA binds to and sequesters FABP7 to the nucleus, resulting in decreased cell migration. We can speculate that DHA could have a different effect in cellular migration activity depending especially on the localization (nucleus or cell membrane) and cellular type (neurospheres or adherent cells).

Preliminary in vivo data corroborate the potential role of FABP7 in the invasion process. Intra-cranial tumors derived from shFABP7 BT165 NS injected cells, showed lower doublecortin expression in parallel to reduction of FABP7 positive cells. In support to this observation in previous studies conducted in our laboratory we observed that in an intra-cranial tumor derived from a highly invasive gliobastoma cell line (BT138 NS), FABP7 expression was significantly higher when compared to a less invasive but highly proliferative cell line (DBTRG NS) (see Figures S3 and S4).

All set of data suggest that FABP7 could be a useful of invasion of glioma cells and that targeting FABP7 could interfere with mechanisms of GBM recurrence.

Inspired by the study of Brun et al. [40], and in order to identify potentially druggable targets that could ‘indirectly’ modulate FABP7 expression, we evaluated the influence of PPAR inhibition on FABP7 expression, as FABP7 expression can be controlled by PPAR [41]. Therefore we investigated the role of modulators such of PPAR δ/γ, PPAR δ, and PPAR γ inhibitors on the expression of FABP7. In this preliminary study, we observed that PPAR (both δ and γ in particular δ/γ) down regulate FABP7 expression.

Interestingly the high expression of FABP7 in irradiated cells decreases significantly after treatment with the PPAR inhibitors and consequently we observed also a reduction in the migrating properties of these cells. Given this data, we suggest that PPAR antagonists might have a potential as anti-migration agents in GBM.

Conventional therapies, such as radiation therapy and chemotherapy, have been used to treat primary brain tumors with modest efficacy, and recent evidence suggests that GBM stem cells are involved in radio−/chemoresistance. Since FABP7 increased after radiotherapy we suggest that this gene could be involved in the process of radio-resistance of these cells.

Targeting FABP7 in combination with chemotherapy could represent a new therapeutic strategy for glioblastoma.

## Supporting Information

Figure S1 **Effect of FABP7 downregulation using other different** ***FABP7*** **siRNA on cellular migration and proliferation.** To exclude the biological off target effect, we also tested the effects of other different *FABP7* siRNAs (siRNA_2 and 7) on cellular proliferation and migration. *FABP7* siRNA treatment resulted in a reduction in proliferation of 30% (±5 SEM) with siRNA_2 and 20% (±5 SEM) with siRNA_7 when compared to scrambled siRNA controls. The effect of *FABP7* down regulation on migration was also investigated using the transwell system. The number of migrating cells after transfection with *FABP7* siRNA_2 and siRNA_7 compared with scrambled siRNA control-transfected cells was significantly (t-test p<0.05) reduced by 30% in BT150 neurospheres.
(TIF)

Figure S2 **In vitro functional role of FABP7 in BT150 NS cells.** Real time PCR performed on BT150 NS cells after the silencing with specific lentivirus particles shows high efficiency of the inhibition (compared to scrambled, Panel A). In this cell line silencing of FABP7 caused an in vitro growth arrest. Ten days after plating shFABP7 NS, cells appeared small and disrupted (compared to scrambled NS, Panel B) or attached to the plate showing signs of differentiation, suggesting that the efficient inhibition of FABP7 expression in this NS line impacts on biological functions.
(TIF)

Figure S3 **Immunohistochemistry analysis of glioblastoma cell lines engrafted into mouse brain.** Photomicrograph of H&E (a, d, g), Ki67 (b, e, h) and FABP7 (c, f, i) stained sections obtained from DBTRG AC-derived (a, b, c), DBTRG NS-derived (d, e, f) and BT138 NS-derived (g, h, i) orthotopic xenografts. Asterisk (*) = Necrotic areas. Arrowheads (>): Pseudopalisading cells. Scale bar = 100 µm.
(TIF)

Figure S4 **Histochemistry analysis of brains from tumor-bearing mice.** Whole brain photomicrograph of Ki67 staining performed in BT138 NS (a) and DBTRG NS (b) generated tumors. In the lower panels are highlighted the different tumor burden of the two tumors. Scale bar = 100 µm.
(TIF)

File S1 **This file includes supporting material, methods and relative references.**
(DOC)
