# Supplementary figures and images for: A Radial Glia Gene Marker, Fatty Acid Binding Protein 7 (FABP7), Is Involved in Proliferation and Invasion of Glioblastoma Cells
Source: PLoS One. 2012 Dec 21;7(12):e52113. doi: 10.1371/journal.pone.0052113 (PMC3528762; doi:10.1371/journal.pone.0052113)

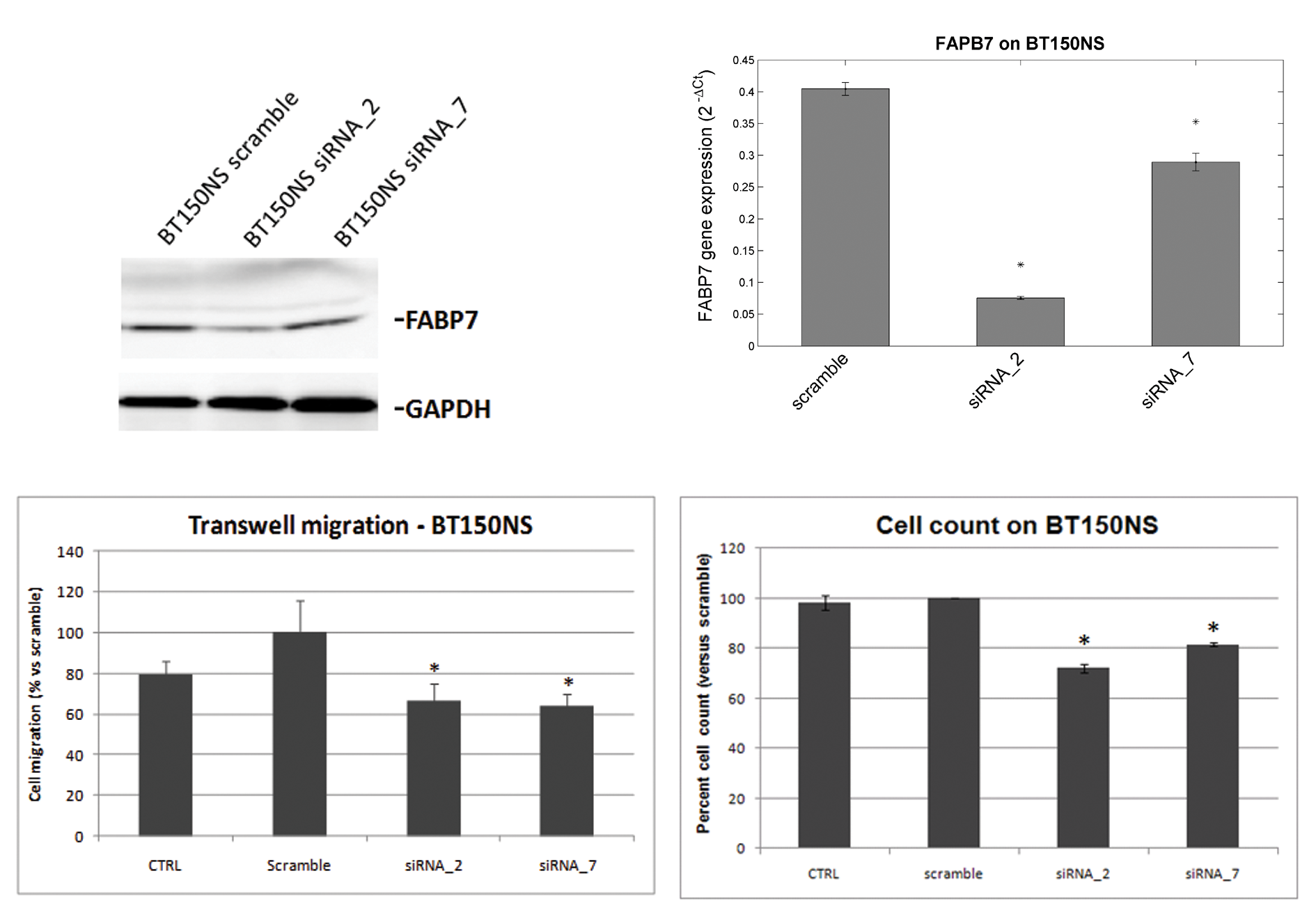

Supplement: Figure S1 — Effect of FABP7 downregulation using other different FABP7 siRNA on cellular migration and proliferation. To exclude the biological off target effect, we also tested the effects of other different FABP7 siRNAs (siRNA_2 and 7) on cellular proliferation and migration. FABP7 siRNA treatment resulted in a reduction in proliferation of 30% (±5 SEM) with siRNA_2 and 20% (±5 SEM) with siRNA_7 when compared to scrambled siRNA controls. The effect of FABP7 down regulation on migration was also investigated using the transwell system. The number of migrating cells after transfection with FABP7 siRNA_2 and siRNA_7 compared with scrambled siRNA control-transfected cells was significantly (t-test p<0.05) reduced by 30% in BT150 neurospheres. (TIF) [file pone.0052113.s001.tif]

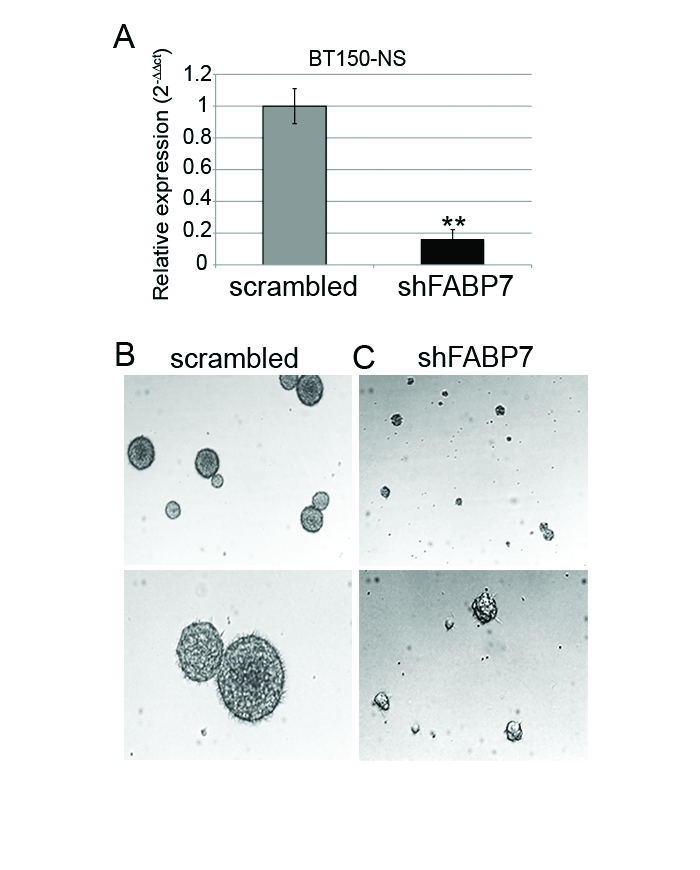

Supplement: Figure S2 — In vitro functional role of FABP7 in BT150 NS cells. Real time PCR performed on BT150 NS cells after the silencing with specific lentivirus particles shows high efficiency of the inhibition (compared to scrambled, Panel A). In this cell line silencing of FABP7 caused an in vitro growth arrest. Ten days after plating shFABP7 NS, cells appeared small and disrupted (compared to scrambled NS, Panel B) or attached to the plate showing signs of differentiation, suggesting that the efficient inhibition of FABP7 expression in this NS line impacts on biological functions. (TIF) [file pone.0052113.s002.tif]

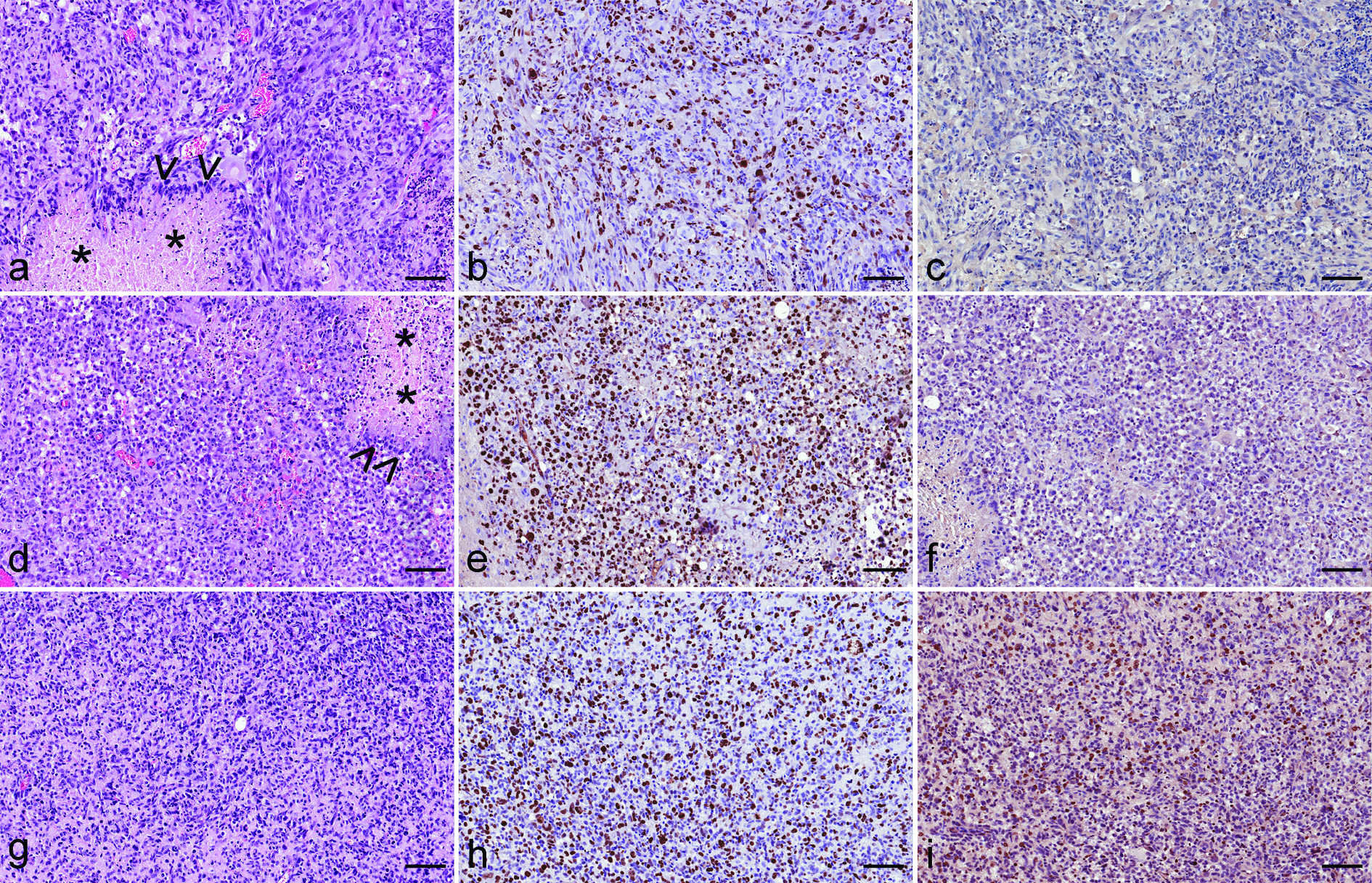

Supplement: Figure S3 — Immunohistochemistry analysis of glioblastoma cell lines engrafted into mouse brain. Photomicrograph of H&E (a, d, g), Ki67 (b, e, h) and FABP7 (c, f, i) stained sections obtained from DBTRG AC-derived (a, b, c), DBTRG NS-derived (d, e, f) and BT138 NS-derived (g, h, i) orthotopic xenografts. Asterisk (*) = Necrotic areas. Arrowheads (>): Pseudopalisading cells. Scale bar = 100 µm. (TIF) [file pone.0052113.s003.tif]

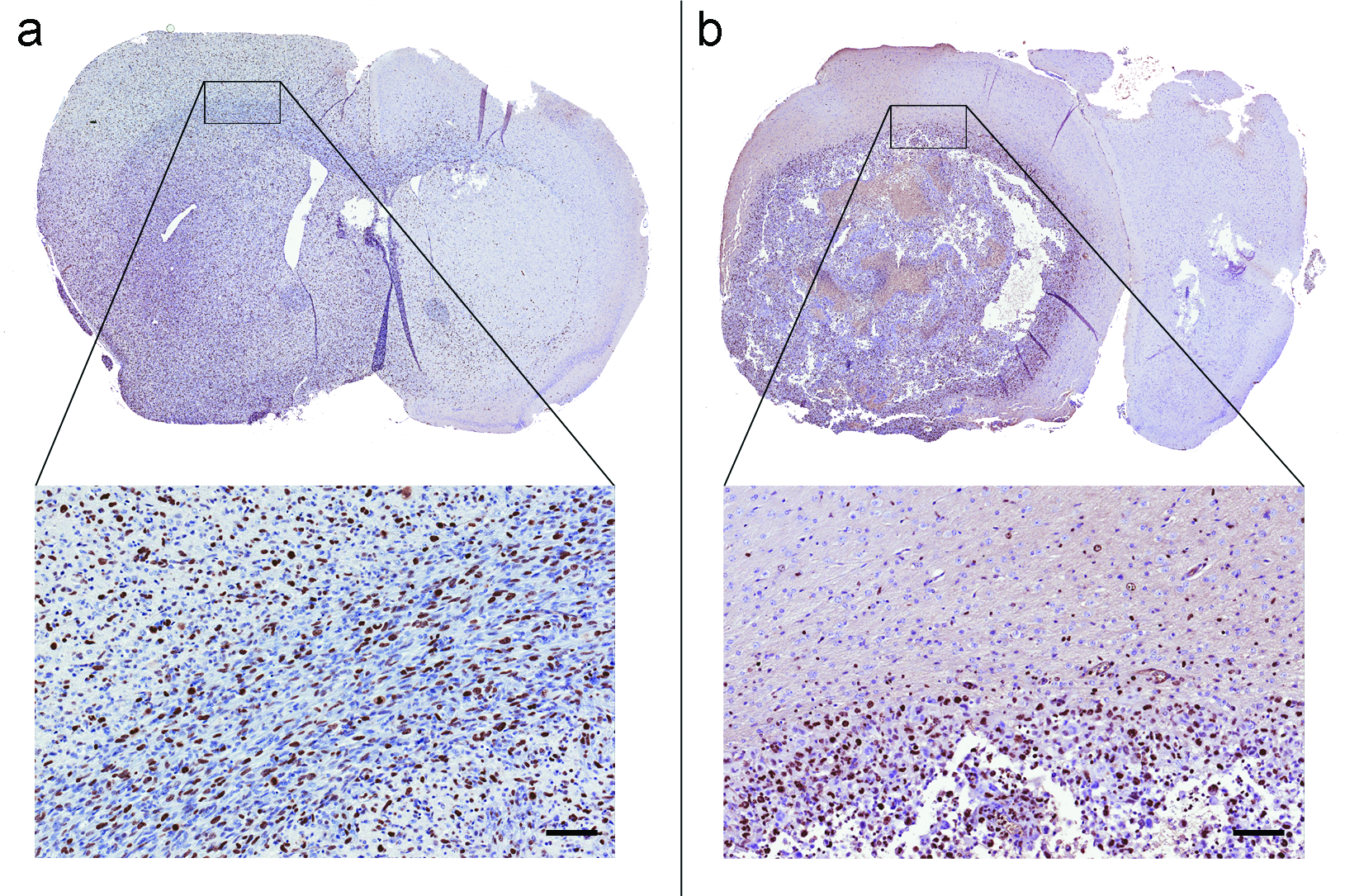

Supplement: Figure S4 — Histochemistry analysis of brains from tumor-bearing mice. Whole brain photomicrograph of Ki67 staining performed in BT138 NS (a) and DBTRG NS (b) generated tumors. In the lower panels are highlighted the different tumor burden of the two tumors. Scale bar = 100 µm. (TIF) [file pone.0052113.s004.tif]
