# Supplementary material for: A Radial Glia Gene Marker, Fatty Acid Binding Protein 7 (FABP7), Is Involved in Proliferation and Invasion of Glioblastoma Cells
Source: PLoS One. 2012 Dec 21;7(12):e52113. doi: 10.1371/journal.pone.0052113 (PMC3528762; doi:10.1371/journal.pone.0052113)
Supplement: File S1 — This file includes supporting material, methods and relative references. (DOC) [file pone.0052113.s005.doc]

**SUPPLEMENTARY MATERIAL**

***Specific effect of the FABP7 down-regulation on cellular migration and proliferation***

In order to demonstrate that the observed reduction of growth and migration of tumor cells (in vitro) transfected with *FABP7* siRNA is specifically due to down-regulation of *FABP7*, and not to a non specific (off-target) effect on cell function, we tested the effect of down-regulation of *FABP7* using two other non-redundant *FABP7* siRNAs.

BT150 NS were transfected with 250 nM siRNAs, the first one targeting the *FABP7* 3’UTR (OligoID: Hs_FABP7_2 Catalog #SI00382564,Qiagen;) and the second one targeting the *FABP7* coding sequence (OligoID: Hs_FABP7_7 Catalog #SI04326623, Qiagen) and negative control AllStars Neg. siRNA Fluorescein (Catalog #1027282,Qiagen) using nucleofector transfection with the AMAXA system (Lonza).

After 48h the silencing efficiency of *FABP7* siRNA was confirmed at the mRNA level (Taqman real-time PCR) and after 72 h at the protein level (by Western blot). As depicted in Figure S1, the silencing capacity of siRNA_2 and siRNA_7 (both at mRNA and protein level) is less prominent than the previously used siRNA (Figure 3). However, the phenotypic effect of these siRNAs is in line with their silencing capacity. The effect of *FABP7* downregulation was then analyzed in proliferation and migration assay.

***Immunohistochemistry of glioblastoma cell lines engrafted into mouse brain***

DBTRG-AC, DBTRG NS and BT138 NS (a de novo highly invasive glioblastoma cell line) were injected orthotopically using the same conditions as reported in the manuscript methods section.

Samples obtained from these cell lines were used for histological evaluation and immunohistochemistry. Brain sections (5-μm) were cut from FFPE samples mounted on Superfrost ULTRA PLUS glass slides (Fisher Scientific, Pittsburgh, PA, USA). After deparaffinization and hydration, the tissue slides were either routinely stained for H&E or processed for Immunohistochemistry (IHC). IHC staining was performed by using a polymer-based detection method. Briefly, slides were placed in a glass container filled with pH 6 citrate buffer and heated in a microwave for 15 minutes for antigen retrieval. Endogenous peroxidase activity was blocked by incubating the slides in phosphate-buffered saline (PBS) containing 3% H_2_O_2_ for 15 minutes. Slides were then incubated in 5% normal horse serum for 30 minutes at room temperature. Primary rabbit monoclonal antibody against Ki67 (dilution 1:100, ab16667, Abcam, UK) or rabbit polyclonal antibody against FABP7 (dilution 1:100, ab27171, Abcam, UK) was applied to slides followed by incubation overnight at 4°C. ImmPRESS Universal Antibody Polymer Detection Kit (ImmPress Reagent Kit, Vector, Burlingame, CA, USA) was applied and the slides were incubated for 30 minutes at room temperature. Between each step of the immunostains, slides were washed three times (5 min/each) in PBS buffer (pH 7.4). DAB substrate (Vector) was used as peroxidase substrate. Sections were thus counterstained with hematoxylin. Slides were scanned with Aperio Scanscope CS (Aperio Technologies, Inc., Vista, CA) and analyzed with Aperio ImageScope viewer (Aperio).

In order to investigate the role of FABP7 in the in vivo tumorigenesis and invasiveness of NS cells, we have analyzed mouse brain xenograft generated by three different cell types. Preliminary in vivo experiments were performed by injecting tumour cells from DBTRG AC, DBTRG NS and BT138 NS into the mouse striatum brain (as above described in details). By hematoxylin-eosin staining, DBTRG NS and DBTRG AC–derived xenografts were morphologically characherized by a dense cellular mass consisting of interlacing bundles of plemorphic, spindle to round cells with occasional giant and multinucleated elements. Cells showed marked anisocytosis, anisokaryosis and bizarre mitotic figures (atypia). All examined cases showed multifocal intratumoral necrotic foci characterized by a serpentine pseudopalisading pattern bordered by karyorectic inflammatory cells, typical of GBM. BT138 NS–derived tumours were morphologically characherized by a densely cellular mass consisting of plemorphic cells with marked signs of atypia. Necrosis was never observed (Figure S3, a, d, g).

As can be appreciated in Figure S3 (b, e) the Ki67 positivity in DBTRG derived tumours supported the suggestion that neurospheres show a higher proliferative activity then their adherent counterpart. Between NS derived tumours, the proliferative index remains comparable. (Figure S3 e, h).

The FABP7 immunostaining was also performed in brain from tumour bearing mice. In the normal part of mouse brain (served as control) we found perinuclear immunoreactivity as reported in literature [1]. In tumours derived from DBTRG AC xenograft, FABP7 positive cells were rare. Immunoreactivity was slightly increased in xenografts derived from DBTRG NS while the highly invasive BT 138 NS derived tumours showed a high presence of immunoreactive cells (Figure S3, c, f, i). This could be related to the high infiltrative behaviour of BT138 NS-generated tumours where tumour cells diffused into the mouse brain without forming a defined tumour mass, as was observed in the DBTRG NS tumours (Figure S4).

**Supplementary references**

1. [Liang Y](http://www.ncbi.nlm.nih.gov/pubmed?term="Liang Y"%5BAuthor%5D), [Bollen AW](http://www.ncbi.nlm.nih.gov/pubmed?term="Bollen AW"%5BAuthor%5D), [Aldape KD](http://www.ncbi.nlm.nih.gov/pubmed?term="Aldape KD"%5BAuthor%5D), [Gupta N](http://www.ncbi.nlm.nih.gov/pubmed?term="Gupta N"%5BAuthor%5D). 2006 Nuclear FABP7 immunoreactivity is preferentially expressed in infiltrative glioma and is associated with poor prognosis in EGFR-overexpressing glioblastoma. Bmc Cancer 6:97.
